# The structural basis for cancer treatment decisions

**DOI:** 10.18632/oncotarget.2439

**Published:** 2014-09-12

**Authors:** Ruth Nussinov, Hyunbum Jang, Chung-Jung Tsai

**Affiliations:** ^1^ Cancer and Inflammation Program, Leidos Biomedical Research, Inc., Frederick National Laboratory for Cancer Research, National Cancer Institute, Frederick, MD 21702, U.S.A.; ^2^ Sackler Inst. of Molecular Medicine, Department of Human Genetics and Molecular Medicine, Sackler School of Medicine, Tel Aviv University, Tel Aviv 69978, Israel

**Keywords:** Cancer treatment decisions, driver mutations, driver genes, redundant pathways, parallel pathways, computational biology, protein structure, cellular network, oncogenic mutations

## Abstract

Cancer treatment decisions rely on genetics, large data screens and clinical pharmacology. Here we point out that genetic analysis and treatment decisions may overlook critical elements in cancer development, progression and drug resistance. Two critical structural elements are missing in genetics-based decision-making: the mechanisms of oncogenic mutations and the cellular network which is rewired in cancer. These lay the foundation for the structural basis for cancer treatment decisions, which is rooted in the physical principles of the molecular conformational behavior of single molecules and their interactions. Improved tumor mutational analysis platforms and knowledge of the redundant pathways which can take over in cancer, may not only supplement known actionable findings, but forecast possible cancer progression and resistance. Such forward-looking can be powerful, endowing the oncologist with mechanistic insight and cancer prognosis, and consequently more informed treatment options. Examples include redundant pathways taking over after inhibition of EGFR constitutive activation, mutations in PIK3CA p110α and p85, and the non-hotspot AKT1 mutants conferring constitutive membrane localization.

## INTRODUCTION

Computational biology tackles cancer in two distinct ways. The first involves analysis of massive quantities of genomic, proteomic, microarray, cell and tissue imaging data produced by experiments, as well as clinical data relating to the tumors, the patients and the results of clinical trials [1–4]. These data are compiled and organized effectively in targeted databases, and diverse software tools are developed to sift through the voluminous compilations to exploit them. The collected data and analyses are immensely important. Databases store vast amount of information, which, if well organized, curated, managed and shared, can produce robust statistical trends. If the number of records is large, they allow correlating basic experimental data with outcomes [5, 6]. However, these are statistical studies; as such, they are not able to provide an insight into why these statistical trends exist and why the correlations are observed. Since each record contains many variables, it is difficult to fully interpret the observed biases. Thus, while enormously useful, this restricts the predictive power for individual patients [7], and hampers personalized drug regimes. The second, complementary way through which computations contribute to cancer research is by revealing the mechanism through which particular genetic or acquired aberration works. To understand mechanisms, and design or computationally screen drugs, structures are needed. The 2013 Nobel Prize in Chemistry underscored the significance of computational structural biology, noting “Computer models mirroring real life have become crucial for most advances made in chemistry today… Today the computer is just as important a tool for chemists as the test tube. Simulations are so realistic that they predict the outcome of traditional experiments” [8]. Further, computations are able to reach a level of mechanistic detail that is hard for experiments to attain. They are able to integrate and interrogate data across a range of scales, introduce and test hypotheses, and most importantly, the results are quantified. Within these active areas of research, here we focus on this second aspect. We aim to put forward what we consider are the major ways through which structural biology, and in particular computational structural biology, contributes to the foundation for cancer treatment decisions. The first step is a definition of cancer: cancer is a breakdown of normal physiological tissue homeostasis due to loss-of-function or gain-of-function. There are multiple ways for each to evolve [9–43].

Cancer can be described by its genetic makeup and expression profiles [44]. This epitomizes personalized medicine, which aims to tailor disease treatment as much as possible to an individual patient [45]. It drives studies of the cancer's genetic features to determine how best to apply the knowledge in the clinic, and calls for genetic testing for inherited cancer risk [46–50]. The complexity of cancer genetics and expression profiles across multiple scales is evident. One example is the histopathological heterogeneity in lung adenocarcinoma which has been classified into subtypes differing in metastatic potential and survival illustrates. No molecular profiles exist to explain these differences. Analysis of discrete areas of the different subtypes, screening for mutations in hotspot regions of the EGFR, KRAS and BRAF genes observed that KRAS and BRAF mutations could be confined to morphological domains of higher grade. However, EGFR driver mutations were observed in all histological subtypes in each tumor. Taken together, this suggests that small biopsies may not adequately represent a tumor's full mutational profile, particularly for later arising but prognostically important mutations such as those in the KRAS and BRAF genes [51]. Despite this, decisions need to address the mutational cause, not the histopathological imaging.

Here our premise is that cancer treatment decisions would benefit from marrying the genetic basis for cancer treatment with the structural basis, which to date has largely been overlooked. The structural basis should be thought of as not only helping to understand the genetics of individual mutations in cancer, but instead as capable of tailoring, innovating and improving the way the disease is treated. Structural considerations argue for a modification of the binary ‘passenger’ and ‘driver’ mutational paradigm. Rather than a mutation classified into one of the two, a ‘passenger’ mutation can become a ‘driver’ if a cooperative mutation takes place during cancer development. Identifying ‘latent driver’ mutations and their expected emergent cohorts in the unstable cancer landscape can inspire more potent personalized treatments.

### Treatment Decisions in the Clinic

Effective personalized cancer treatment requires information. Much of it is available and known to be crucial; some of the rest may be compiled but hidden in the massive amount of data generated by cancer genome sequences, their analyses and patients' genetic tests. Among the key problems facing physicians are the enabling technology and the time to diagnose actionable findings in the patient's genome – as compared to cancer genomes – and clinical data that will improve treatment decisions for the patient; in addition looms the question whether the actionable data have been a priori identified as such. Current cancer treatment decisions largely rest on identification of the patient's driver gene and driver mutations through comparisons to those determined statistically across populations of patients. Ideally, a cancer diagnostic platform should help the oncologist to rapidly translate all relevant and available clinical, molecular, and drug data into effective treatment choices for individual patients. However, diagnostic platforms generally assume that actionable driver mutations have been unraveled, and that these are the ones that appear most frequently in cancer. A mutation is classified as either a driver or a passenger. If the latter, it is of no interest to the treating oncologist. By contrast, here our premise is that actionable mutations (and thus also proteins) should be thought of as not only those diagnosed as drivers, but also some of those presumed to be passengers. A passenger mutation may have no observable clinical effect; however, combined with another presumed passenger mutation, or some external factor (like infection) it can become a driver. Such pre-existing ‘latent driver’ mutations may additively lead to an outcome resembling driver mutations. Diagnosis of the patient's actionable pre-existing latent driver mutations may arm the oncologist with foreknowledge of cancer progression, resistance and more effective drug combinations. Since the mutational environment differs across a patient and cancer population, such a prognostic foresight can lead to more targeted patient-specific therapies.

Oncologists are faced with the challenge of deciphering molecular data and determining the treatment implications. Each tumor presents a distinct set of genetic aberrations among which are driver and latent driver mutations. These can influence the drug's mode of action and its aftermath. The latent drivers' concept calls for reevaluation of genetic decision platforms which are exploited by physicians and powered by high end computing. Because identification of latent drivers rests on residue combinations, larger statistics will be required posing a major challenge.

### An Overview of Cancer: the Preeminent Role of the Cellular Network

Homeostasis provides an intricate balance of cell proliferation and death. Tipping the scale toward cell growth causes cancer. A normal cell is transformed into a tumor cell primarily through alterations in genes regulating cell growth and apoptosis. These involve oncogenes with gain-of-function and tumor suppressor genes with loss-of-function, respectively. Oncogenes promote cell growth and survival. Activating mutations in the Ras protein at codons 12, 13, and 61 [52, 53] promote oncogenesis. In contrast, tumor suppressor genes arrest cell division or induce apoptosis. Cancer typically results from genetic alterations involving a combination of uncontrolled growth and failed anti-proliferative cellular responses, underscoring the involvement of the cellular signaling network. That the cellular network is chief player is also indicated by experiments: genome-wide analysis of DNA copy number and somatic mutation frequencies in melanoma BRAF data [54] revealed significant differences. These allowed classification into four groups with 70% accuracy, indicating that distinct genetic pathways result in distinct melanoma cancer types. Another example concerns PI3K, whose link to cancer is among the strongest in the cell [55, 56]. PI3K integrates growth and survival signals from RTKs and Ras to the mTOR, MAPK, FOXO1 and GSK3β signaling pathways. Class I PI3Ks have a regulatory subunit (p85 α, β, or γ) and a catalytic subunit (p110 α, β, or γ). Constitutive activation of the PI3Ks in the p110α subunit is observed in 30% in the human breast, colon and endometrial cancer patient population. A subpopulation (~10%) of colorectal cancer patients presents inactivating mutations in the p85 regulatory subunit. While imidazopyridine-based drugs J124 and J128 strongly inhibited growth *in vitro* in the nM range as well as in medulloblastoma cell lines, only modest effects on tumor growth inhibition were observed *in vivo*. These data beg the question of which PI3Kα-dependent processes impact the spread of the primary tumor. Thus, linking genetic alterations to signaling pathways in cancer cells is the very first step toward targeted therapeutic development [57], as well as in battling drug resistance [58]. Cancer cells exploit redundant pathways to overcome drug action.

Cancer is a complex genetic disease. Hannahan and Weinberg formulated a useful framework consisting of a set of common capabilities acquired by cancer cells through mutagenesis [59]. These include sustaining proliferative signaling, evading growth suppressors, resisting cell death, enabling replicative immortality, inducing angiogenesis, activating invasion and metastasis. These hallmarks reflect genome instability, which generates genetic diversity, and inflammation. Recently they extended the concept of cancer biology by including two enabling characteristics of reprogramming energy metabolism and evading immune destruction [60]. Cancer cells achieve these abilities mainly by rewiring existing cellular programmes that normally take place during development to coordinate cell proliferation, migration, polarity, apoptosis, and differentiation during embryogenesis and tissue homeostasis. The Darwinian character of cancer cells confers the ability to proliferate and survive through random mutations and epigenetic changes followed by selection of resistant variants [61] under circumstances that would normally be deleterious.

### Types of Cellular and Molecular Events Leading to Cancer

Cancer-causing scenarios are diverse; they include genetic and epigenetic changes in cancer cell genomes. Genetic events cover large scale aberrations in the genome such as gain-of-function or loss-of-function mutations, deletions, fusions, rearrangements and gene duplications, such as CNVs (copy number variations) [62–66]. Tumor suppressor genes may be deleted and regions harboring oncogenes may be amplified, as for example for p16 and myc, respectively [67, 68]. Rearrangements, inversions and translocations, can result in tumor-driving fusion products as in the case of BCR-Abl [69–71] and the Philadelphia Chromosome [72, 73] as well as fusion events in solid tumors [74, 75]. Epigenetic alterations do not involve changes in the nucleotide sequence [76–78]. They influence gene expression through chromatin reorganization and gene accessibility, via alteration of DNA methylation patterns, silencing, posttranscriptional regulation of signaling molecules by microRNAs, histone modifications, alternative RNA splicing, and more. Epigenetics can work through over- or under- gene expression. Cancer-causing scenarios similarly include over/under protein degradation. All bypass normal growth controls.

Computational cancer biology focuses on analyzing molecules and processes that play a major role in cancer, including those above. It uses genome-scale measurements (genomic, proteomic, and metabolomic) to assemble models of cellular processes and disease which can provide blueprints of normal and diseased cell functions. Methods often rely on high-throughput data. They aim at relationships between molecular characteristics of cells, such as how do genome aberrations and changes in copy number, a result of increased genome instability in cancer, affect gene expression as well as elements such as miRNAs, and how the changes affect the function of related proteins. Considerable research focuses on biomarkers, at the genome, transcriptome or proteome levels that are prognostic of cancer progression or predictive of response to specific therapeutic agents [59, 60, 79]. Interpreting copy number data, the effect of genome changes on the transcriptome and proteome level profiles, epigenetic changes, somatic evolution, gene sets in specific cancer types, mutational landscape statistics, such as The Cancer Genome Atlas (TCGA), and data that measure the effects of drugs are major strategies in computational cancer biology.

Collectively, these embrace diverse areas of research. Below, we address key questions in cancer biology that relate to structure. From the standpoint of structural biology, two problems stand out: the mechanisms of mutations and deciphering the network that governs cellular response under physiological conditions and its rewiring in cancer. The examples below underscore the limitations of an approach based solely on genetics, and the significance of complementing it by structural insight in making treatment decisions.

### Cancer Cell Signaling Deregulates the Cellular Network

Proliferation can result from upstream or downstream deregulation of signaling. Upstream signaling can be perturbed by overexpression of growth factors [80, 81], elevated levels of receptors at the cancer cell surface, somatic mutations in the receptors that result in structural changes that facilitate ligand-independent firing, and constitutive activation of proteins in signaling pathways downstream, which bypass ligand-mediated receptor activation. One example is Protein kinase C (PKC) which activates the MEK-ERK pathway, independent of Ras activation and dependent on Raf [82]. Downstream mutations are also common: 40% of human melanomas contain mutations in the B-Raf protein, constitutively activating signaling through the mitogen-activated protein (MAP)-kinase pathway [83]. Another example concerns drug resistant mutations in Raf's catalytic domain leading to Ras-independent Raf dimerization [84]. Cancer typically involves co-occurring mutations.

**Figure 1:**
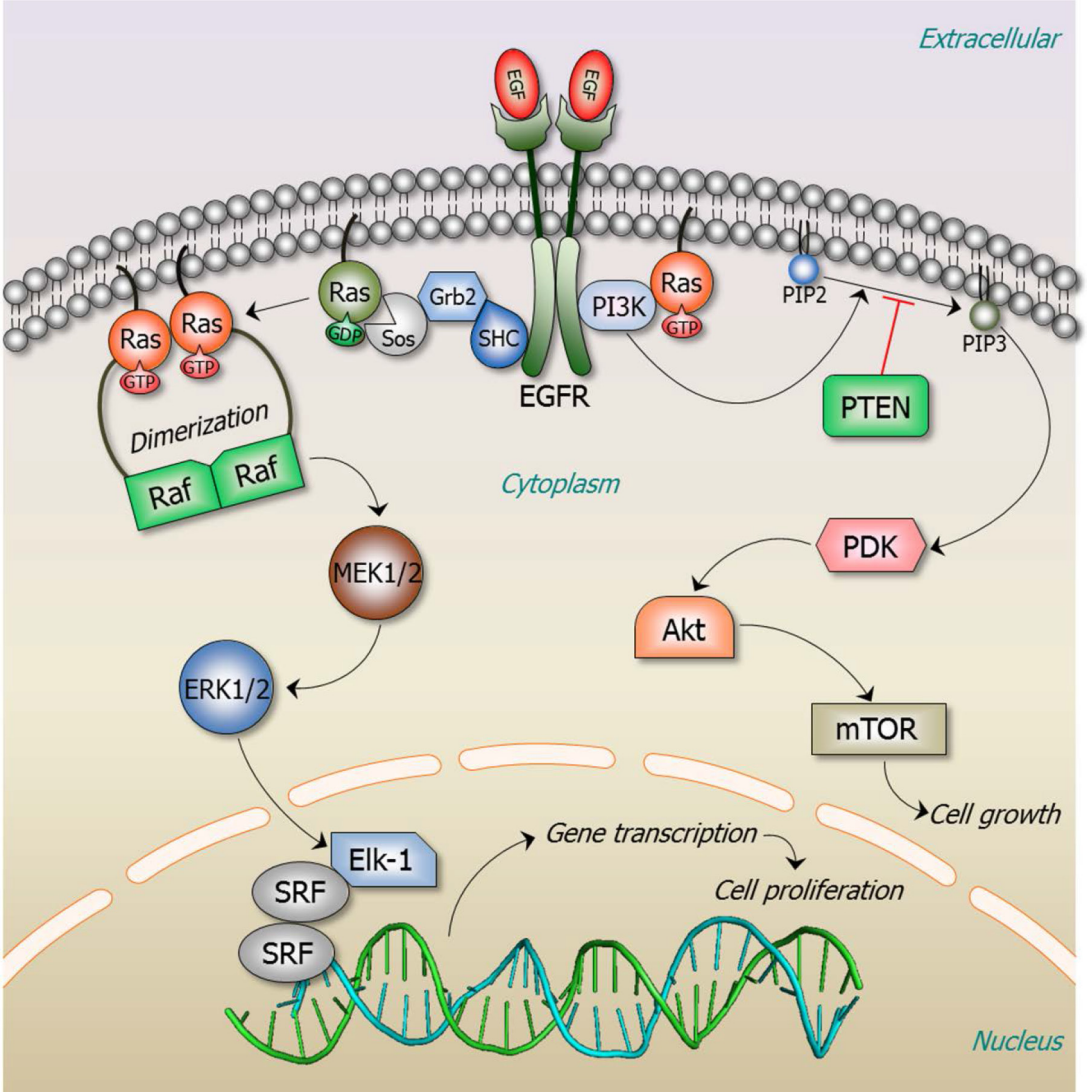
Ras signaling pathways Ras signaling is involved in numerous cellular functions, including cell proliferation, apoptosis, migration, fate specification, and differentiation. A key Ras effector pathway is the mitogen-activated protein kinase (MAPK), Raf-MEK-ERK pathway. EGF binds to the extracellular domain of the epidermal growth factor receptor (EGFR), a receptor tyrosine kinase (RTK). The signal is transmitted through the transmembrane domain resulting in EGFR dimerization and activation. Activated EGFR recruits the son of sevenless (SOS), a guanine nucleotide exchange factor (GEF), to its phosphorylated C-terminal tail via the adaptor proteins, SH2-adaptor protein (SHC) and growth factor receptor-bound protein 2 (Grb2). GEF exchanges GDP by GTP, activating Ras. Active, GTP-loaded Ras dimerizes and binds Raf, thereby promoting Raf dimerization and activation. Active Raf dimer phosphorylates and activates mitogen-activated protein kinase kinase 1 and 2 (MEK1/2), which induces ERK1/2 activation. Transcription factor Elk-1 is among ERK1/2 many downstream phosphorylation targets. Elk-1 binds to its cofactor, a dimer of serum response factor (SRF), leading to transcription activation and cell proliferation. Active GTP-bound Ras regulates a number of signaling pathways; among these is phosphatidylinositol 3-kinase (PI3K). PI3K is a heterodimer with a regulatory (p85) and catalytic (p110) subunits (not shown here). RTKs recruit the p85 subunit of PI3K. Ras activates p110 independently of p85 [172]. PI3K phosphorylates phosphatidylinositol-4,5-bisphosphate (PIP2) to phosphatidylinositol-3,4,5-trisphosphate (PIP3), a process which can be reversed by the action of phosphatase and tensin homologue (PTEN). PIP3 recruits Phosphoinositide-dependent kinase-1 (PDK1) that phosphorylates a serine/threonine kinase, Akt (also known as PKB, protein kinase B) in the plasma membrane. This further induces the activation of mammalian target of rapamycin (mTOR) complex, one of the major pathways leading to cell growth. This pathway plays important roles in Ras-mediated cell survival and proliferation.

Deregulation typically takes place by interfering with molecular checkpoint switches which control the transitions between and within cell cycle phases. Under normal conditions, multiprotein switches generate robust transitions and trigger stable oscillations. The scales are tipped to cancer when the switch is abolished [57]. To capture the protein in its physiological environment, we consider proteins as nodes in the cellular network. Their switches are controlled by two inherent and related factors: the network motif and the signal integration mechanism. One example of the effect of network motif concerns the PI3K/Akt/mTOR pathway (Fig. 1). PTEN, a tumor suppressor gene, negatively regulates the pathway through its lipid phosphatase activity. It counteracts PI3-kinase by degrading its phosphatidylinositol (3, 4, 5) trisphosphate (PIP3) product, thereby keeping a check on cell growth and proliferation. Loss-of-function PTEN mutants, or underexpression due to promoter methylation fail to dampen the signaling flux and are observed in several cancers [85]. However, use of small molecule inhibitors of this pathway remains limited due to the presence of compensatory feedback loops such that inhibition of one molecule often leads to activation of another resulting in chemoresistance. Via negative feedback mTOR activation inhibits PI3K proliferative signaling. The inhibition (e.g. by rapamycin) increases PI3K/Akt/PKB activity, crippling its antiproliferative effects [86, 87]. Another example is provided by the Ras protein, an activator of a number of signaling pathways, including Ras/Raf/Mek/Erk (Fig. 1). Oncogenic Ras mutations (involving G12, G13 and Q61) abolish the GTP→GDP hydrolysis reaction, retaining Ras in a constitutive GTP-loaded active state [88].

### Cancer Can Hijack Signal Integration

Each protein (node) in the cellular network receives concomitant signals from more than one source, e.g. phosphorylation and a binding event, multiple post-translational modifications (PTMs), or two or more binding events. Each event is an allosteric effector. The recipient protein node integrates the incoming signals and transmits an output response, which reflects its resultant conformational distribution. The mechanisms of signal integration and output response are expressed by proteins forming or quenching interactions [89]. There are three possible mechanisms to switch a node from an OFF state to an ON state [89, 90]: (i) incremental activation by a graded switch, with full activation requiring all events. Src kinases, which belong to the nonreceptor tyrosine kinase family, can provide an example. Activation takes place through dephosphorylation of the C-terminal tail, phosphorylation of the activation loop, binding of a ligand to the SH2 domain, and binding of ligands to the SH3 domain. Each event leads to a higher level of activity. (ii) AND-gate, an all-or-none switch with only two types of responses: inactivity or full activity. All signaling events are required for activation. The Tec family kinases, where both phosphorylation of the loop and intramolecular binding of the SH2-kinase linker to the kinase are required for activation provide an example. (iii) OR-gate, an all-or-none switch with two types of responses, where one set of events is sufficient for full activation. Syk tyrosine kinase activation, which takes place by either phosphorylation in the SH2-kinase linker or by binding of phosphorylated immunoreceptor tyrosine-based activation motifs (ITAMs), provides an example. The presence of both stimuli does not enhance the kinase activity beyond each stimulus alone. Structural data on the ON- and OFF- states are required to annotate the logic gate type. However, in the absence of experimental data, assigning the logic gate type is challenging. Oncogenic and drug resistance mutations interfere with native signal integration resulting in loss of control. To constitutively activate a node in the cellular network, oncogenic mutations are likely to be more frequent and effective in the OR gate than in the Graded and AND gates, with single (or fewer) mutations hijacking control.

Oncogenic and drug resistance mutations can hijack the signal integration mechanism resulting in loss of control. One example relates to deregulation of the Ras-Raf-Mek-Erk pathway by mutations in B-RAF. Raf activation requires formation of an asymmetric dimer configuration. Under normal physiological conditions, Ras-GTP-membrane recruitment, dephosphorylation of Ser259, and dissociation of 14–3–3 result in stabilization of an open conformation of Raf monomer, which favors dimer formation. Gain-of-function mutations in the B-RAF gene, the most oncogenic of which is B-RAF^V600E^, induce ERK activity independently of the normal EGFR-Ras signals [84, 91–93], by allosterically shifting the population [94–98] of the ensemble toward dimer-favored monomer conformation. Vemurafenib treatment results in deletion mutant p61B-RAF^V600E^ with enhanced dimerization and activity through a similar allosteric mechanism. It is also possible that binding of the drug to one p61B-RAF^V600E^ monomer inhibits drug binding to the other, resulting in one active monomer per dimer and abolishing drug resistance.

### Key Questions from the Structural Standpoint

The model of cancer argues that the first consideration is that the experimental data include information on whether the malignancy is the outcome of (i) over- or under expression; if overexpression it can be reflected for example in a higher gene copy number or differential regulation of gene expression. (ii) Normal expression, however with gain- or loss-of-function mutations. Most oncogenic mutations result in gain-of-function; loss-of-function mutations can take place in repressors with a similar outcome. (iii) Aberrant protein degradation. The first step involves verification of the oncogenic mutation through available structural data; if absent, via high quality modeled structures. This allows approaching questions such as what is the mechanism of the oncogenic mutation on the protein conformational level and how it affects its interactions [99–101] which are the oncogenic ‘driver’ versus ‘passenger’ mutations and why [102]; why a specific mutation can have a more profound effect when the protein is bound to a specific effector as in the case of Raf influencing Ras' intrinsic hydrolysis; how the mutations affect the circuitry and its rewiring in different types of cancers and under different (individuals, tissues, cell types) conditions; and related to this why specific mutations may activate specific pathways; which are the isoform-specific pathways, and how do these work. Further, cancers often involve multiple different clones impeding therapeutics; do these collaborate and if so how.

**Figure 2:**
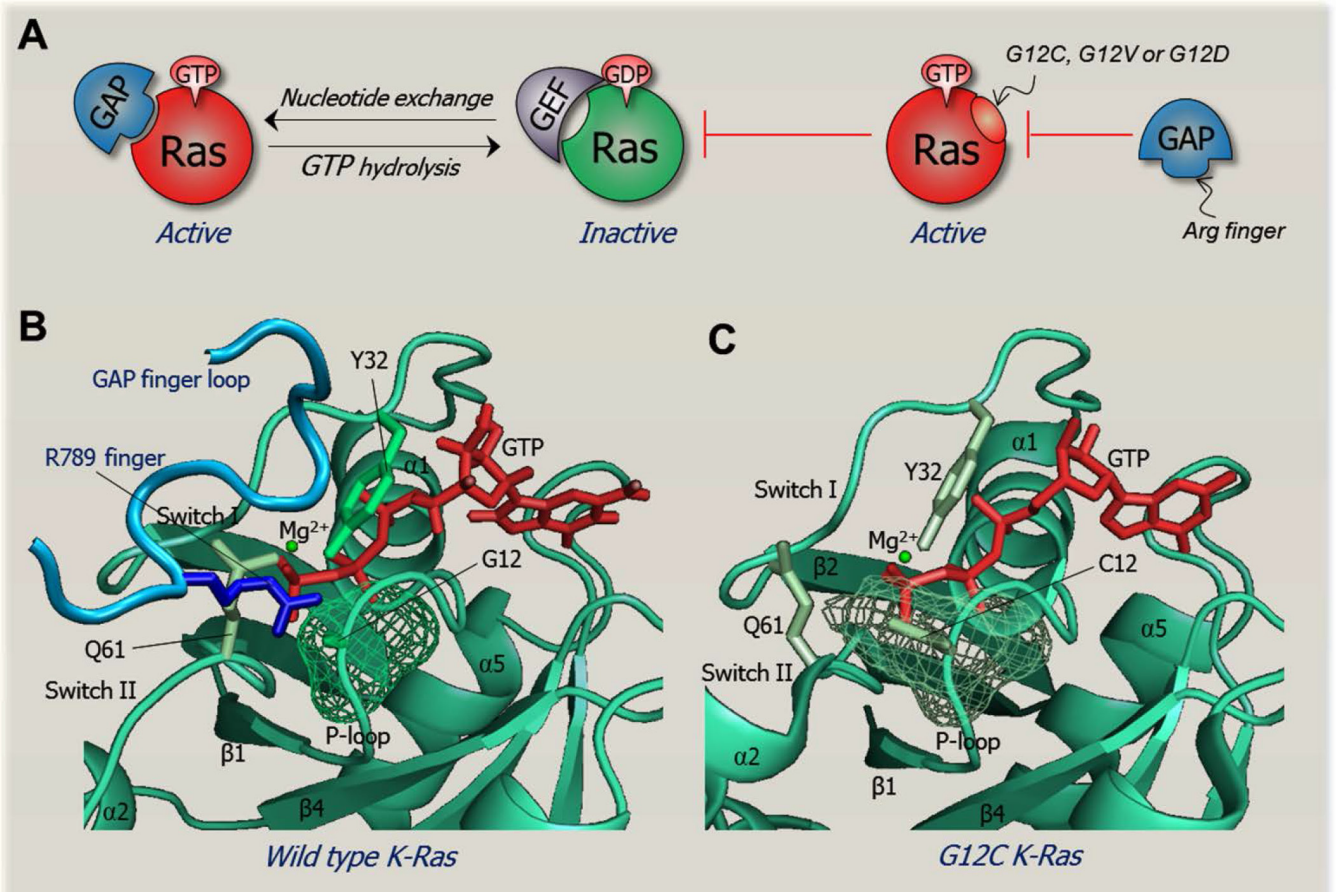
The structural basis of an oncogenic mutation The figure illustrates how an oncogenic mutation can work on the structural level. Ras the most common mutated oncogene in cancer is shown as an example. Wild type Ras acts as a binary signal switch cycling between active and inactive states. Ras only binds its effectors in its GTP-bound active state. Ras is activated by the son of sevenless (SOS) nucleotide exchange factor (GEF). In contrast, the GTP→GDP hydrolysis, helped by GTPase-activating protein (GAP) inactivates Ras. A key oncogenic mechanism aborts the hydrolysis reaction, keeping Ras in a constitutively active GTP-bound state. Residues most prone to these mutations are G12, G13 and Q61. Mutation of G12 K-Ras is most prevalent and oncogenic in colon cancer [173]. G12C and G12V K-Ras mutants activate the Ral guanine nucleotide dissociation stimulator (RaLGDS) pathway, whereas G12D preferentially activates the PI3K and MAPK signaling pathways [105, 106]. The reason for this differential preferred activation is still unclear. Figure **(A)** sketches Ras regulation under normal conditions (on the left hand-side) and constitutive activation (right hand-side). The constitutively active conformation of Ras harboring these mutations does not permit formation of the transition state required for catalysis upon binding to GAP. Under normal conditions, the flexibility of G12 allows the Arg789 side-chain (Arg finger) on the finger loop of GAP to insert into Ras active site. However, G12 mutants with bulkier or charged residue prevent the Arg finger insertion, blocking the transition state with GAP [174]. It is also likely that these mutations allosterically differentially affect the effector binding sites (not shown). **(B)** This panel illustrates native Ras in complex with GAP, poised for the catalytic reaction. The crystal structure of GDP-H-Ras/RasGAP complex (PDB code 1WQ1) is remodeled with the GTP-K-Ras crystal structure (PDB code 3GFT). The finger loop of RasGAP is in blue (taken from the complex, PDB code 1WQ1). The Arg finger is highlighted as a blue stick, positioned at the Ras active site, near the G12 residue in green mesh. **(C)** This panel clarifies why mutation of G12 prevents hydrolysis through a steric clash mechanism in which the G12C residue in green mesh prevents the insertion of Arg finger. Crystal structure of G12C GTP-H-Ras mutant (PDB code 4L9W) is remodeled to G12C GTP-K-Ras mutant.

A mutation can constitutively activate (or repress) by stabilizing the active (or destabilizing the inactive) state, as in the case of the EGFR [103, 104]. Alternatively, it may block a reaction, as in the case of Ras G12, G13 and Q61 mutations which hamper GTP hydrolysis, thus retaining Ras in a constitutively activated state (Fig. 2). Nonetheless, why G12C and G12V K-Ras mutations in lung adenocarcinoma preferentially activate the Ras' RalGDS pathway, whereas G12D prefers the Raf/mitogen-activated protein kinase (MAPK) and PI3K pathways [105, 106] is still unclear. One possibility is that allostery plays a role [104, 107–110]. Besides their direct effects, these mutations are established to induce allosteric effects at the effector binding site. Q61 is a key allosteric residue [111, 112], as is Y64 [113]. Another question concerns the mode of the specific inhibition of K-Ras4B, a highly oncogenic splice variant of the K-Ras isoform, by calmodulin and its enigmatic signaling consequences. To understand the mechanism of oncogenic mutations requires conformational detail, achievable with the help of molecular dynamics simulations. EGFR provides remarkable example [103, 104, 114–116]. However, questions such as why mutations in certain isoforms which are highly similar in sequence and structure are much more frequent than in others may not necessarily relate to protein conformations; instead the answer may lie in over- (under-) expression as could be in the case of K- versus H- and N-Ras [117, 118], underscoring the importance of the three types of data above.

Identifying the location of the mutations and their stabilizing/destabilizing actions may provide clues to their ‘driver’ or ‘passenger’ properties [119–123]. High-throughput somatic missense mutations detected in tumor sequencing can be mapped onto structures to provide first-hand information. In protein-protein interfaces they may abolish a specific interaction or enhance it [124, 125]. Conformational analysis may distinguish between tumorigenic ‘driver’ mutations from their neutral passenger counterparts.

**Figure 3:**
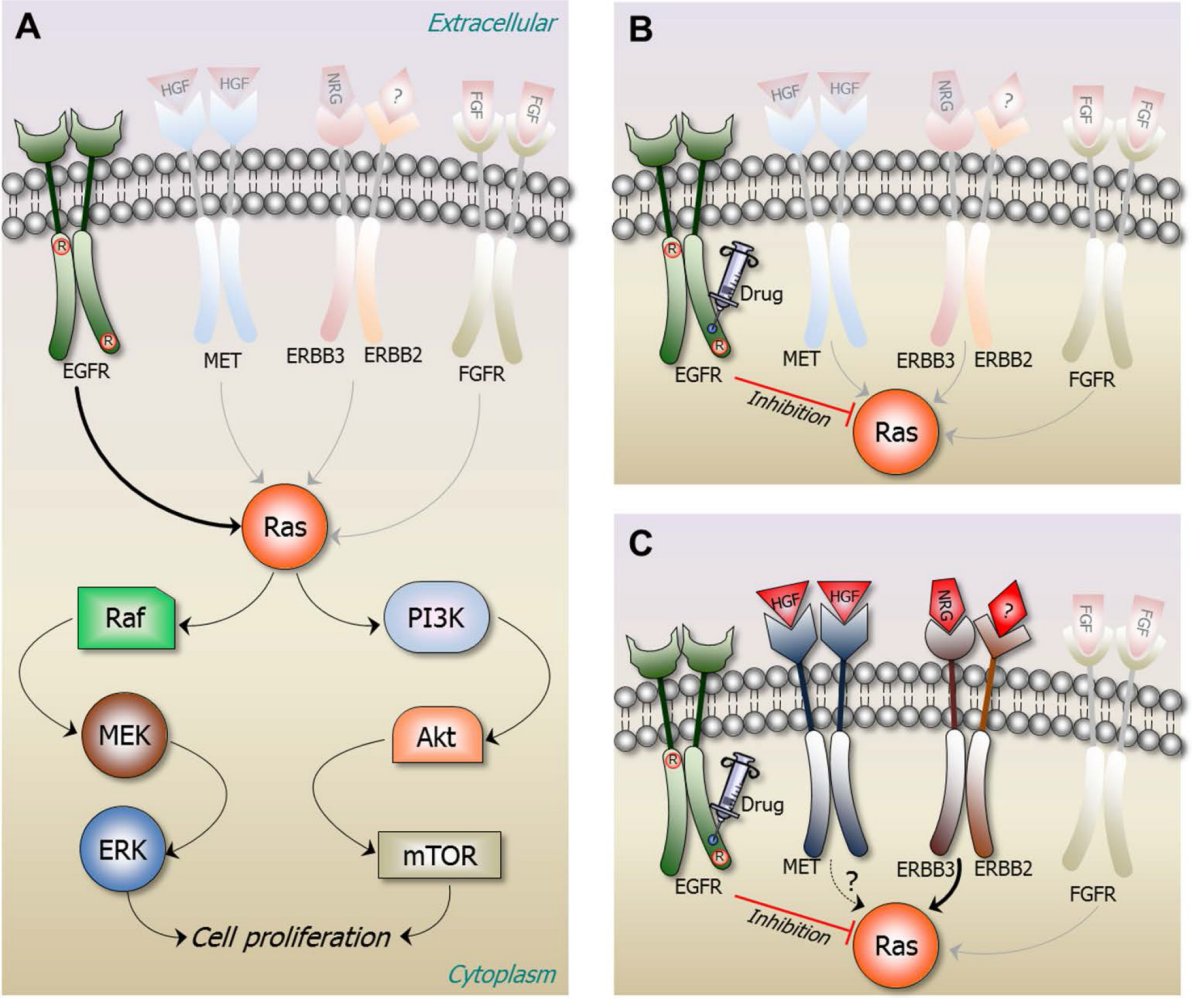
A structural view of redundant pathways taking over during drug resistance Ras is normally activated in response to the binding of extracellular ligands to various receptors. Among these is epidermal growth factor (EGF) binding to its cognate receptor EGFR, as shown in Figure 1. Upon EGF binding to the extracellular domain of EGFR, the intracellular domain of EGFR forms an asymmetric dimer in the cytosol. EGFR and its ERBB receptor family members can form homo- or hetero-dimers. Downstream signaling proceeds through Ras in the Raf-MEK-ERK and/or PI3K-Akt-mTOR pathways. The figure provides a sequence of events induced by a constitutive mutation taking place in EGFR, keeping it in an active state even in the absence of its ligand. Drug treatment abolishes EGFR signaling; however, a drug resistant mutation leads to overexpression of another receptor, populating an otherwise low-activity second receptor. **(A)** L858R mutation in EGFR kinase (the circled R) causes non-small cell lung cancer (NSCLC) by constitutively activating its kinase domain [175]. Under normal conditions EGFR largely populates its inactive state. The mutation shifts the free energy landscape of EGFR stabilizing its active with respect to its inactive conformation even in the absence of a bound EGF. **(B)** Drugs such as the 4-anilinoquinazolines gefitinib (Iressa) [176] and erlotinib (Tarceva) [177] can inhibit the activity of EGFR L858R mutant. **(C)** However, tumors develop resistance, in this case one possibility is through overexpression of MET [178]. Overexpressed MET leads to phosphorylation of ERBB3 which interacts with ERBB2. The ERBB2/ERBB3 receptor can activate Ras and thus its PI3K-Akt signaling pathway, independent of EGFR. A key question is how the blockage of an addicted growth pathway is able to rewire the oncogenic cellular network within a short period, leading to MET's overexpression and ERBB3 activation.

The single most confounding question is how a cancer cell escaping drug treatment is able to rapidly adapt and rewire its network toward uncontrolled growth. Another, on a different level, is how proteins escape degradation. Tumor cells avoid apoptosis and promote survival in a number of ways [126–128]; among these is loss of TP53 tumor suppressor function, up-regulating antiapoptotic (Bcl-2, Bcl-xL) or down-regulate proapoptotic (Bax, Bim, Puma) regulators, aborting the extrinsic ligand-induced death pathway and interfering with programmed cell death by shifting the balance in favor of survival [129]. They may also evade the immune surveillance. Inflammation can support the tumor microenvironment [130]. Constructing the complex structural pathways [131–137] would further aid therapeutic targeting to mitigate drug resistance [58]. Negative feedback loops can recoil cancer. For example, there are many ways to activate Erk through receptor tyrosine kinases (RTKs), T-cell receptors (TCRs), and GPCRs. Blocking EGFR may lead to other pathways taking over (Fig. 3). Figuring out the network and its robust regulatory motifs and circuitry requires structural data. Given its sheer complexity and its multiple cell- and tissue-wide effects, this is perhaps the paramount challenge facing structural biology.

### Redundant Pathways and Therapeutics

Deliberately targeting specific proteins has been a major strategy. The emergence of drug resistance fostered an alternative, involving drug combinations either for the same protein or to a lesser extent different proteins albeit usually in the same pathway [138]. Two strategies can further expand the repository. The first, ‘allo-network drugs’ can bolster the repertoire of targets with proteins that can work by transmitting the effects to target proteins in the pathway allosterically across protein-protein interfaces [139–142]. The second accounts for redundant pathways. Compiling a repertoire of combinations of redundant pathways and targets within them would facilitate systematic evaluation. A sufficiently robust compilation can uncover and stockpile ‘drug cocktails’ [58] to alleviate the almost-inevitable relapse.

Growing experimental evidence supports the partially redundant signaling pathways (Fig. 3). Thus, a targeted therapy inhibiting one pathway in a tumor may not completely shut off cancer cell survival. Surviving cancer cells adapt to the therapy through mutations, epigenetic reprogramming, or remodeling of the stromal microenvironment, eventually provoking renewed tumor growth, abrogating the drug's efficacy. Since the number of parallel signaling pathways supporting a given function can be expected to be limited, targeting all of these therapeutically, through a strategy such as the one described above can be achievable. However, while in principle possible, compiling the repertoire of parallel pathways accounting for negative feedback loops is a yet another highly challenging task confronting structural biology [58].

### Outline of the Components of the Strategy

Mapping guidelines implementing the structural basis for treatment is not straightforward. Broadly, two elements are needed: (i) a detailed structural model of the oncogenic driver mutations and (ii) a repertoire of redundant pathways in the cell to allow judicious ‘drug cocktail’ combinations.

For the first, via sequence alignments the mutation can be identified. Molecular dynamics simulations can verify its mechanism. EGFR provides a good example [57]. The kinase catalytic domain is allosterically activated by formation of an asymmetric dimer in which the C-lobe of the ‘activator’ kinase domain interacts with the N-lobe of a second ‘receiver’ kinase domain [143]. In contrast, in symmetric dimers, both kinases are in inactive conformations. Unliganded EGFR fluctuates between the monomer and dimer states [144]. Structural data indicated that in normal cells the kinase domain of EGFR is mostly populated either in a stabilized autoinhibited monomer or in an inactive symmetric dimer, making the formation of the asymmetric dimer possible, but not favorable. EGF binding, induces a conformational change in the extracellular domain which facilitates the kinase asymmetric dimer association. Oncogenic mutations in the juxtamembrane region that stabilize the dimer (such as Δ746–750 or L858R) can either destabilize the inactive conformation and/or stabilize the active conformation as shown by simulations [114].

For the second, which pathways to combine (to avoid alternative routes by drug resistant mutations, Fig. 3) and which protein targets to select within these (to minimize toxicity) present a daunting challenge. An organized redundant pathway resource based on structural data can be a first step toward a ‘pathway drug cocktail’ [58]. The National Cancer Institute has published a summary of drug combinations, as well as common combinations for colon and rectal cancer. 5,000 combinations of 100 existing cancer drugs have been tested. High-throughput screening techniques led to 150 drugs that were genotype-selective for Ras or B-Raf mutations. These were searched for pairs that could inhibit metastasized melanoma. While such exhaustive strategies can obtain beneficial results, a strategy based on more structural information of the cellular network may be expected to allow for more accurate and deliberate targeting of specific cancers at a fraction of the cost.

Finally, structural data can be critical in elucidating mutations that on their own are ‘passengers’; however, when combined become ‘drivers’. To date, a mutation has been classified as either a ‘driver’ or a ‘passenger’. However, the AND and Graded logic gate integration mechanisms discussed above (but not OR) argue that allosteric mutations can work like allosteric binding events cooperating to constitutively activate (or, inactivate in the case of a repressor) a cellular network node [89]. We reason that whether a mutation is a ‘passenger’ or a ‘driver’ is likely to depend on the mutational landscape of the protein. This is in line with successive stepwise cancer development and progression [145–150]. Pre-existing latent mutational population can combinatorially merge with other newly acquired genetic alterations. This can be tested experimentally and statistically. In principle, structural analysis should be able to annotate the mutational data and identify ‘latent driver’ mutations. Forecasting which existing ‘passengers’ will turn into ‘drivers’ and upon which mutational changes could establish more powerful treatments.

### Intrinsic and Extrinsic Influence

Redundant pathways can be intrinsic or extrinsic. At a higher level, the signaling circuitry describes the intercommunication between multiple distinct cell types that collaborate to progressively form malignant stages of cancer. It also includes signaling between microbiota and the human cell. Recent data indicate the tight linkage, arguing for studies of a metaorganism at the structural regulatory level.

Compelling evidence points to tumors as highly heterogeneous populations derived from a common progenitor [151]. The multiple cancer cell clones and combinations of co-existing mutations in the same cell including splicing variants of the same isoform [152], may be the reason why current pharmaceutical strategies involving targeted magic bullets toward a specific protein and mutation not only fail but may incite more resistant aggressive cells. By accounting for populations of cells, the complex dynamic circuitry, and population shifts in response to changing conditions, computational structural biology may foment and chart new territory [153]. Even living things must conform to the laws of quantum mechanics and structural chemistry.

### The Structural Basis for Improving Decision Platforms for the Clinic

Treatment decisions are based on data and its interpretation. Data sources, types and volume increase rapidly. This is particularly the case in cancer. Not only is there a rapid increase in the number of sequenced cancer genomes across a population, but in addition, the mutational rates and the microenvironment evolve rapidly. The NIH has recognized the need for assembling and organizing platforms for ‘big data’ – and within such a framework, cognitive capabilities that ‘understand’ the context, uncover answers, and continuously learn from experiences – in the battle against cancer. The unprecedented breadth and depth of clinical oncology data and knowledge guide informed decisions. However, extracting actionable insights from this information still poses significant challenges. Beyond the valuable day-to-day patient care data and clinical trials – often trapped in disparate and remote databases and established routines for identification of key genomic factors – there is a need to step back and reconsider basic premises. It is clear that the identified driver mutations in genomic cancer screens are of paramount importance. The question is – are we certain that mutations labeled as passengers are expendable? Biology has long taught us that it does not follow a binary ‘Yes’ or ‘No’ definition; conditions matter and these work by shifting the expression of mutations on the structural – and thus functional – level. A mutation can be classified into a driver, latent driver which can be expressed in combination with other mutation, and true passengers. Because latent driver mutations pre-exist in the cancer mutational load, their detection equips the oncologist with a mechanistic perception, permitting prediction of the potential cancer evolution. A comprehensive gene testing to evaluate genetic changes in a patient's tumor can thus help oncologists to more effectively manage treatment options.

Early on most tumors were treated according to what they looked like under the microscope; over the past decade focus shifted to the molecular reasons for why cancer grows. There is a consensus that informed cancer medicine can help clinicians tailor anticancer treatment to individual patient tumor characteristics. This represents a significant shift in the ability to understand, and respond to, vast amounts of ‘big data’ and may have enormous potential to improve decision making for health care. Here we take a further step toward forecasting tumor evolution. Our thesis is that the basis of actionable mutations is indeed the informatics-definition platforms and the associated clinical trials; however, that definition should improve to account for the latent driver repository and redundant signaling pathways in cancer prognosis and drug cocktail treatment regimes. It is difficult to estimate the percentage of patients that would benefit from improved decision platforms incorporating latent drivers. Even though it can be low compared to the high frequency of driver mutations – in cancer treatment decisions they cannot be overlooked.

### Examples of Low Frequency Mutations Acting as Drivers

Two recent examples related to the AKT1 gene illustrate that low frequency mutations can act as drivers mimicking the same structural mechanisms, underscoring the need to reconsider decision strategies based solely on cancer genome statistics. Mutational activation of the PI3-kinase-Akt-mTOR pathway is the most frequent oncogenic event in breast cancer. The hotspot AKT1 E17K mutation occurs in approximately 3% of primary breast cancers. The mutation confers constitutive plasma membrane localization in the absence of growth factor stimulation, leading to increased Akt1 activation and phosphorylation of downstream target proteins. Functional analysis of large scale breast cancer sequencing studies identified six non-hotspot AKT1 pleckstrin homology domain mutants. Three of these cause constitutive activation of Akt1 [154]. Of note, like the hotspot E17K mutation, these mutants confer constitutive membrane localization of Akt1. These same three mutants also showed oncogenic activity in a cellular transformation assay. The other three mutants were inactive. These findings not only validate novel driver mutations in AKT1 and extend the number and type of mutations that activate the PI3-kinase pathway in human breast cancers, they also point out that genetics-based identification of driver mutations is incomplete. Non-hotspot, lower frequency mutations are not necessarily passengers, and may act via similar mechanisms. A second study discovered somatic mutations at the pleckstrin homology (PH) domain-kinase domain (KD) interface. These mutations abolish the interactions between the domains which are essential for maintaining AKT in an inactive state. These AKT1 somatic mutants are constitutively active, leading to oncogenic signaling. Also, the AKT1 mutants are not effectively inhibited by allosteric AKT inhibitors, in agreement with the requirement for an intact PH-KD interface for allosteric inhibition [155]. Allosteric drugs emerge as an advantageous therapeutic strategy due to their higher specificity and thus lower toxicity [156].

## CONCLUSIONS

Personalized treatment decisions are fraught with bottlenecks. The genetic basis for cancer treatment rests on knowledge of the personal cancer mutation spectrum [157] and its comparison with known statistical trends across primary and metastasized tumors. However, distinguishing between driver and passenger mutations is difficult and it is still unclear how many mutations are active at any given stage of a tumor.

Consideration of pathways through which resistance can take place is not straightforward either. Pathways may appear simple and linear; however, this is not the case, particularly not in the complex circuitry of cell survival, differentiation, growth and death. That a simple pathway diagram is not good enough can be seen from the Raf dimer example [158–160]. Pathways are also poorly understood at the level of detail required to shut them down effectively. Coupled to this is the deployment of the immune system. Innate immunity pathways are intimately linked to proliferative signaling, energy metabolism, angiogenesis, invasion, and survival pathways, sharing major cellular circuits [124, 161–167].

The structural basis for cancer treatment decisions focuses on unveiling the mechanism of the mutation on the conformational level and deciphering the redundant pathways that can be rewired in drug resistant mutants to increase the likelihood of avoiding relapse. Decision making rests on therapy combinations based on this information. ‘Latent driver’ mutations can be thought of not only as bearing on the question of why common adult tumors, such as pancreatic, breast and brain cancer, often have three to six mutated genes while several tumors have only one or two driver gene mutations [168], but also on how the blocking of an addicted growth pathway is able to rewire the oncogenic cellular network within a short time period in drug resistance.

Cancer research encompasses phenotypic complexities; however, these may manifest a small set of underlying organizing principles [60]. Computational structural biology is a powerful quantitative science. It combines biology and chemistry/physics of single molecules and their interactions in atomic detail and on a large scale. Together with experiment and statistical ‘big data’ genetic and clinical analysis, it may help in laying the foundation for new paradigms in the biological sciences to elucidate the basis of cancer and abate malignant transformations. Combining the structural basis, the genetic basis and clinical data can revitalize personalized treatment regimes.

Matching targets for selective cancer therapy is difficult [169, 170]. Nonetheless, recently strategies have been proposed to restrict the combinatorial space, minimize toxicity, and increase the precision and power of such restrictive combinations, altogether leading to drugs that could be tested in clinical trials [171]. Leveraging the enhanced identification of drug targets, including repertoires of redundant pathways combinations, may be helped by such innovative concepts.
